# The Role of PI3k-Gamma Modulation in Bacterial Infection: A Review of the Literature and Selected Experimental Observations

**DOI:** 10.3390/antibiotics14030315

**Published:** 2025-03-18

**Authors:** Daniel Sun, Alexandria Hoffman, Fatemeh Askarian, Elisabet Bjånes, Eric X. Lin, Judith Varner, Victor Nizet

**Affiliations:** 1 Skaggs School of Pharmacy and Pharmaceutical Sciences, UC San Diego, La Jolla, CA 92093, USA; das033@health.ucsd.edu; 2 Department of Pediatrics, UC San Diego, La Jolla, CA 92093, USA; ashoffma@umn.edu (A.H.); faaskarian@health.ucsd.edu (F.A.); ebjanes@health.ucsd.edu (E.B.); erlin@health.ucsd.edu (E.X.L.); 3 Biomedical Sciences Graduate Program, UC San Diego, La Jolla, CA 92093, USA; jvarner@health.ucsd.edu; 4 Moores Cancer Center, UC San Diego, La Jolla 92093, USA

**Keywords:** host–pathogen interactions, drug repurposing, PI3k, *Staph aureus*

## Abstract

**Background:** Phosphoinositide 3-kinase is a potent target for cancer therapy due to its significant role in the regulation of cellular growth and proliferation. Dysregulation of the PI3k signaling cascade can constitutively activate growth pathways to trigger the progression of cancer, resulting in the development of multiple inhibitors as cancer therapeutics. **Objectives**: The wide array of cells expressing PI3k also include immune cells, and the inhibition of these receptors has shown promise in combating inflammation and infectious disease, a relationship we sought to examine further. **Methods**: We infected wild-type and PI3kγ knockout murine macrophages as well as PI3kγ inhibitor-treated THP-1 human macrophage-like cells with *Staphylococcus aureus* and quantified inflammation through gene expression analysis, protein secretion assays, and immunofluorescence imaging. **Results**: We observed that knockout of PI3kγ in murine macrophages alongside pharmacological inhibition through IPI549 treatment in THP-1 cells led to an NF-κB-driven suppression in transcription and release of inflammatory cytokines upon infection with methicillin-resistant *Staphylococcus aureus*. We were also able to confirm that this suppression of NF-κB translocation and subsequent decrease in inflammatory cytokine release did not compromise and even slightly boosted the bacterial killing ability. **Conclusion**: PI3k is primarily targeted for cancer therapies, but further exploration can also be carried out on its potential roles in treating bacterial infection.

## 1. Introduction

As one of the largest fields of biomedical research, cancer receives billions in funding annually to discover new chemotherapeutics and improve treatment outcomes [1]. A novel pathway that is under investigation as a chemotherapeutic target is phosphoinositide 3-kinase (PI3k). PI3k, an enzyme activated by G protein-coupled receptors or receptor tyrosine kinases, initiates the PI3k/Akt signaling axis and regulates multiple growth pathways, including the mammalian target of rapamycin (mTOR) [2]. While primarily associated with growth and proliferation, PI3k’s highly conserved nature and influence on immune pathways have also made it a target of interest in infectious disease research. With 700,000 deaths annually attributed to antibiotic-resistant microbes and projections of as many as 10 million deaths per year by 2050, antibiotic resistance presents an urgent need for alternative treatments to reduce infection-associated morbidity and mortality [3]. Due to the high expression of specific isoforms in immune cells and their important roles in immune function, PI3k is a promising target for further study to modulate the host immune response [4,5,6,7].

## 2. Phosphoinositide 3-Kinases

The American Cancer Society projected 609,820 cancer deaths and 1,958,310 new cases in 2023 [8]. Cancer development often involves amplified receptor activity or uncontrolled enzyme activity, leading to excessive phosphorylation and dysfunctional growth pathways. The convergence of these pathways at PI3k makes it an efficient target for inhibition [2]. Mutations throughout the PI3k/Akt pathway are implicated in cancer development, with mutations in PI3k alone being linked to breast, lung, gastric, kidney, and colorectal cancers [9]. Given its critical role in cancer progression, it is not surprising that the PI3k/Akt pathway is often activated across various tumor types, making it a key focus for drug development [10].

**Figure 1:**
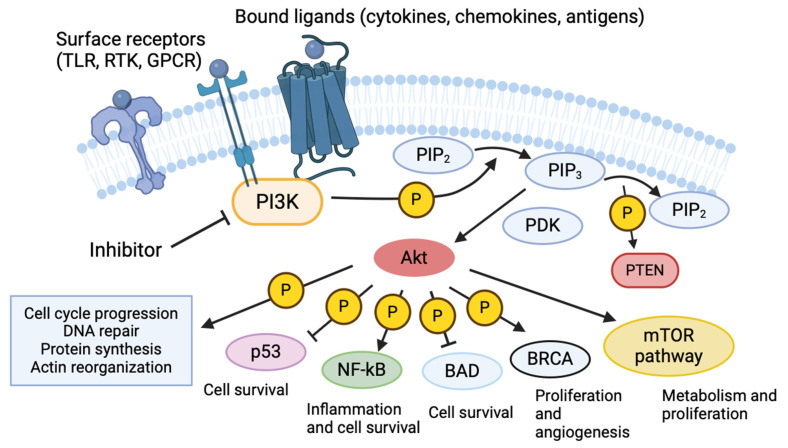
Overview of PI3k pathway. PI3k is activated by various surface receptors, including toll-like receptors (TLRs), receptor tyrosine kinase (RTK), and g-protein coupled receptors (GPCRs). The kinase domain phosphorylates phosphatidylinositol biphosphate (PIP2,) converting it to phosphatidylinositol triphosphate (PIP3). PIP3 subsequently phosphorylates Akt, which serves as a messenger to initiate several metabolic pathways, several of which are illustrated above. PI3k plays an important role in regulating cell survival and proliferation, actin reorganization, and inflammation.

Mammalian PI3k is divided into three classes (I, II, III) comprising eight isoforms, all of which share similar membrane-binding C2, helical, and kinase domains on their catalytic subunits [11]. Class I kinases are further subdivided into α, β, γ, and δ isoforms. The α and β isoforms are ubiquitously expressed across most cell types, while the γ and δ isoforms are predominantly found in myeloid cells [12,13]. PI3k is activated by various cell surface receptors, including receptor tyrosine kinases (RTKs), Ras GTPases, and G protein-coupled receptors (GPCRs) [13]. Ligand activation of these receptors prompts the PI3k regulatory subunit to facilitate the kinase activity of the associated catalytic subunit [14]. The catalytic subunit utilizes ATP to phosphorylate phosphatidylinositol lipids, thereby triggering downstream signaling cascades [11]. This kinase activity is countered by a phosphatase and tensin homolog (PTEN), which tightly regulates the cellular levels of phosphatidylinositol-3,4,5-triphosphate (PIP3) and its precursor, phosphatidylinositol-4,5-bisphosphate (PIP2) [15]. PIP3 localizes to the plasma membrane, where it enables phosphoinositide-dependent kinase-1 (PDK-1) to phosphorylate protein kinase B (Akt), modulating processes including cell survival, apoptosis, metabolism, DNA repair, and motility [15]. Some notable pathways that are affected by Akt and, by extension, PI3k include cell survival and inhibition of apoptosis through NF-κB, metabolism and translation through mammalian target of rapamycin (mTOR), DNA repair through p53, and reactive oxygen species generation through NADPH oxidase (Figure 1) [16,17]. These are also pathways where aberrant signaling and dysregulation can lead to cancer development.

Drug development has historically focused on Class I kinases due to their well-characterized roles in cancer [15,18]. As a result, multiple PI3k inhibitors have been approved or are currently in clinical trials, targeting either all isoforms of PI3k together (e.g., copanlisib) or specific isoforms (e.g., alpelisib, eganelisib) (Table 1) [18]. These inhibitors are designed to mimic and outcompete ATP at the kinase binding site, blocking Akt phosphorylation and preventing the cascade of growth processes that are disrupted or overactive in cancer [19]. LY294002, a non-selective PI3k inhibitor derived from the flavonoid quercetin, is widely used in PI3k screens due to its high stability [20,21,22]. Similarly, wortmannin, a metabolite isolated from *Penicillium wortmannii*, is another non-selective inhibitor that is frequently used in PI3k studies and will be discussed in this review [23]. IPI549 (eganelisib), a PI3k inhibitor that is specific to the γ isoform (Class I), was developed by Infinity Pharmaceuticals as an anti-tumor therapy aimed at reprogramming myeloid cells and will be discussed in the selected experiments presented later in this manuscript [24].

## 3. PI3k in Infectious Disease

In 2019, approximately 13.7 million deaths, accounting for over 15% of the global death rate, were attributed to infections, with 54.9% being linked to just five bacterial pathogens [31]. Three of these pathogens—*Staphylococcus aureus*, *Pseudomonas aeruginosa*, and *Klebsiella pneumoniae*—are part of the ESKAPE group (*Enterococcus faecium*, *S. aureus*, *K. pneumoniae*, *Acinetobacter baumannii*, *P. aeruginosa*, *Enterobacter* spp.), which are notorious for their virulence and antibiotic resistance [32]. The rise in antimicrobial resistance, coupled with the stagnation of antibiotic discovery, has ushered in the “Resistance Era”, underscoring the urgent need for novel approaches to combat infections [33]. One promising avenue involves repurposing approved drugs that are not currently indicated for infections. Even without direct antimicrobial effects, these drugs can influence the host–pathogen interface by enhancing or protecting host immune cells or by sensitizing bacteria and inhibiting their virulence [34]. While tumor and infection environments differ, they share common cytokines and cell types that are involved in inflammatory responses [35]. PI3k also plays a role in both conditions, as bacteria can exploit host PI3k enzymes to spread or evade immune detection [36,37,38]. This overlap highlights the potential of further exploring PI3k in disease treatment. The γ and δ isoforms of PI3k, enriched in lymphoid and myeloid cell populations, are particularly significant in immune responses [39,40]. Recent studies in cancer and chronic inflammatory disease models have uncovered key roles of the PI3Kγ isoform in macrophage polarization, myeloid cell trafficking, wound healing, and fibrosis [24,41,42,43,44,45,46].

The epithelial layer serves as the first barrier to infection, making it an ideal starting point for investigating the role of PI3k in host–pathogen interactions (Table 2). The bioterror threat pathogen *Bacillus anthracis* relies on spore internalization for dissemination upon inhalation. However, treatment with the pan-Class I PI3k inhibitors wortmannin and LY294002 significantly attenuated spore internalization by blocking the actin activity that is required for spore entry into epithelial cells [47]. *Chlamydia trachomatis*, the most common bacterial sexually transmitted infection, manipulates the PI3k pathway to prevent apoptosis in infected HeLa cells. This effect was reversed with LY294002, which restored pro-apoptotic protein function [48]. Similarly, HeLa cells are vulnerable to invasion by the neonatal pathogen group B Streptococcus, but this invasion was significantly disrupted with LY294002 treatment [49]. *S. aureus* internalization in endothelial cells was linked to Akt phosphorylation, with upstream PI3k inhibition by LY294002 and wortmannin abolishing bacterial entry [50]. The Gram-negative nosocomial pathogen *P. aeruginosa* also activates the PI3k pathway in epithelial cells during infection, and PI3k blockade reduces bacterial internalization [51]. *Helicobacter pylori*, the causative agent of gastric ulcers, triggers persistent PI3k/Akt pathway activation in gastric epithelial cells, leading to increased reactive oxygen species production. This damaging process was blocked by LY294002 or the antioxidant N-acetyl cysteine [52]. Thus, while epithelial cells serve as a critical first line of defense, they can also be manipulated by pathogens to enhance virulence.

If a pathogen breaches the epithelial layer, it encounters immune cells such as neutrophils, where PI3k plays a key role in regulating apoptosis, motility, and tissue infiltration [53]. Neutrophils employ multiple forms of programmed cell death to maintain homeostasis and support innate defense. These include apoptosis, which reduces intracellular niches for pathogen replication, and the release of neutrophil extracellular traps (NETs), which ensnare bacteria in an antimicrobial meshwork [54]. As a regulator at the top of multiple signaling cascades, PI3k mediates several anti-apoptotic effects in neutrophils. These include increased transcription of anti-apoptotic proteins such as Mcl-1, Bcl-2, and Bcl-xL, as well as the inhibition of death caspases and cytochrome C release from mitochondria [55]. In PI3kγ-deficient mice, neutrophils show increased apoptosis under both basal and LPS-stimulated conditions, attributed to reduced activation of NF-κB and CREB and decreased levels of Mcl-1 and Bcl-xL [55]. The zoonotic pathogen *Francisella tularensis* hijacks and delays neutrophil apoptosis, but treatment with LY294002 accelerates apoptosis and restores homeostasis during infection [56]. Similarly, multiple *Chlamydia* species inhibit neutrophil apoptosis via IL-8 and PI3k/NF-κB activation, an effect that is reversed by LY294002 treatment [57,58]. PI3kγ also facilitates the invasion of the foodborne pathogen *Campylobacter jejuni*. In mice treated with the PI3kγ inhibitor AS252424, bacterial counts in the spleen and mesenteric lymph nodes were significantly reduced due to decreased neutrophil overactivity and migration [59]. In zebrafish infected with *P. aeruginosa*, neutrophil infiltration and motility in response to injury were reduced by LY294002, which inhibited PI3k activity, which is essential for tail contraction and actin polarity [53,60]. Given its central role in regulating apoptosis and cellular motility, PI3k acts as both a mechanism for bacterial immune evasion and a promising therapeutic target (Table 2).

Similarly to bacteria, multiple viruses exploit the anti-apoptotic capabilities of PI3k to prolong host cell survival, thereby enhancing viral replication and release [61,62,63]. Recent studies on SARS-CoV-2, the virus that is responsible for COVID-19, and other coronaviruses suggest that PI3K/mTOR inhibitors hold therapeutic potential [64,65]. Building on research into the role of PI3k and IPI549 in cancer, a collaboration at our university uncovered beneficial effects of these inhibitors in SARS-CoV-2 infection models and severe bacterial infections [42,66]. Lung tissue from COVID-19 patients was found to overexpress the gene for PI3kγ. When the PI3k inhibitor IPI549 was administered to Syrian golden hamsters infected with SARS-CoV-2, it significantly reduced neutrophil accumulation and lung inflammation [67]. RNA sequencing in ACE2 transgenic C57BL/6 mice treated with IPI549 revealed downregulation of inflammatory cytokine expression. Similarly, IPI549 improved survival in aged BALB/c mice infected with SARS-CoV-2. Further studies demonstrated that IPI549 treatment enhanced lung healing and vascularization. Experiments using PI3kγ knockout C57BL/6 mice replicated many of these effects, showing reduced inflammation and immune cell recruitment in various respiratory disease models [67]. In systemic infection with methicillin-resistant *Staphylococcus aureus* (MRSA), PI3kγ knockout mice exhibited improved survival rates and reduced serum IL-1β levels and maintained bacterial loads and phagocytic activity that were equivalent to wild-type mice [67]. Moreover, in murine cells stimulated with LPS, IPI549 treatment led to reductions in inflammatory markers such as TNF and IL-6. These findings highlight the critical role of PI3k in modulating inflammation during infectious diseases, supporting its potential as a therapeutic target for both viral and bacterial infections (Table 2).

## 4. PI3k Regulation of Macrophage Responses to Infection

Macrophages, key components of innate immunity, serve as a nexus for interactions and receptors that regulate inflammation and the immune response [68]. The role of PI3k in macrophage activity is complex, with evidence supporting both pro-inflammatory and anti-inflammatory effects. Lipopolysaccharide (LPS), a component of Gram-negative bacterial cell membranes, is widely used to stimulate macrophages via Toll-like receptor (TLR)-4 activation and is a major mediator of sepsis [69,70,71]. PI3k inhibitors, such as wortmannin, have been shown to increase nitric oxide (NO) and TNF production in LPS-stimulated murine macrophages [72]. In human monocytic cells, PI3k inhibition similarly enhanced TNF and tissue factor (TF) expression in LPS-stimulated THP-1 cells by increasing the activation of the downstream transcription factors Egr-1, AP-1, and NF-κB [73]. This pro-inflammatory phenotype was further reflected in PI3kγ-knockout macrophages, which exhibited increased NF-κB release when stimulated by LPS [42]. These findings collectively suggest that inhibiting the PI3k/Akt pathway enhances inflammatory responses in TLR-stimulated macrophages [74].

Conversely, some studies have linked PI3k inhibition to the suppression of inflammatory pathways, primarily through its regulation of NF-κB, a key promoter of pro-inflammatory gene transcription [75]. In BV2 microglial and RAW264.7 murine macrophage cells, LPS-induced NF-κB binding activity was attenuated by LY294002 treatment [76,77]. Similarly, in human macrophages, LY294002 significantly reduced the LPS-induced production of IFN-γ, a critical cytokine for antimicrobial defense and activation of the innate immune system [78,79]. Wortmannin also inhibited LPS-induced nitric oxide (NO) production, producing effects that were akin to direct NF-κB inhibition in microglial cells [80]. Additionally, natural compounds like pinocembrin and sophoraflavanone G were shown to reduce PI3k phosphorylation and decrease the production of inflammatory markers, including IL-1β and TNF, in macrophages [81,82]. These findings suggest that PI3k/Akt pathway inhibition can effectively control inflammation. However, the effects of PI3k signaling appear to depend on specific stimuli and the TLR pathways that are involved, potentially explaining conflicting reports regarding its role in macrophage function [83]. Further investigation into PI3k’s regulation of immune responses is particularly warranted in the context of live bacterial infection.

Studies on bacterial pathogens such as *H. pylori* have demonstrated that PI3k regulates actin cytoskeleton rearrangement during bacterial phagocytosis in murine macrophages, an effect that is attenuated by PI3k inhibitors such as wortmannin and LY294002 [84]. *Legionella pneumophila*, which replicates within macrophages, showed up to an 80% reduction in invasion when J774A.1 macrophages were treated with LY294002 or wortmannin, or when PI3k was genetically disrupted [85,86]. In group B *Streptococcus* (GBS)-infected THP-1 cells, PI3k inhibition reduced actin projections, phagocytic uptake, NF-κB nuclear localization, and intracellular bacterial survival while increasing macrophage cell death [87]. Similarly, in *P. aeruginosa* infections, LY294002 reduced phagocytosis by over 80% [88]. However, PI3k inhibition can also impair the innate immune response. For instance, in *Streptococcus pneumoniae* infections, reduced alveolar macrophage recruitment following PI3k inhibition led to decreased lung bacterial clearance and survival [89]. In the context of *S. aureus* infection, PI3k inhibition reduced autophagy and phagocytosis while increasing NF-κB-mediated cytokine production [90]. These findings suggest that the role of PI3k in macrophage responses varies depending on the pathogen and cell type, influencing both pro-inflammatory and anti-inflammatory outcomes (Table 2).

**Table 2:** The effect of PI3k inhibition on various cell types in the context of bacterial and viral infections or lipopolysaccharide (LPS) stimulation.

Cell Type	Stimulant	Effect	Reference
A549 (Epithelial)	*Bacillus anthracis*	Blocks actin activity and attenuates spore internalization	[47]
HeLa (Epithelial)	*Chlamydia trachomatis*	Restores pro-apoptotic functionality	[48]
	*Group B Streptococcus*	Reduces bacterial internalization	[49]
MDCK (Epithelial)	*Pseudomonas aeruginosa*	Reduces bacterial internalization	[51]
GES-1 (Epithelial)	*Helicobacter pylori*	Inhibits bacteria-induced PI3k overactivation and excessive reactive oxygen species production	[52]
BEC (Endothelial)	*Staphylococcus aureus*	Reduces bacterial internalization	[50]
Human Neutrophils	*Francisella tularensis*	Restores homeostatic apoptotic functions during infection	[56]
	*Chlamydia pneumoniae,* *C. psittaci*	Reverses infection-induced delay of apoptosis	[57,58]
Murine Neutrophils	LPS	Increases apoptosis	[55]
	*Campylobacter jejuni*	Reduces migration and infiltration	[59]
Zebrafish Neutrophils	*Pseudomonas aeruginosa*	Reduces motility and infiltration to site of infection	[60]
Various Murine and Hamster Cells	SARS-CoV-2	Downregulates inflammatory cytokine expression, improves survival, and reduces immune cell recruitment	[67]
Human Monocytes	LPS	Reduces production of IFN-γ	[78]
THP-1 (Human Monocytic Cell Line)	LPS	Enhances TNF and TF expression	[73]
	Group B *Streptococcus*	Reduces actin projections, phagocytic uptake, and NF-κB localization	[87]
Murine Macrophages	LPS	Increases nitric oxide and TNF production	[72]
	LPS	Increases release of NF-κB from inhibitory complex	[42]
	LPS	Enhances TNF, IL-6, and TF expression	[74]
	*Helicobacter pylori*	Blocks internalization of bacteria	[84]
	*Streptococcus pneumoniae*	Reduces macrophage recruitment, lung bacterial clearance, and survival	[89]
RAW264.7 (Murine Macrophage Cell Line)	LPS	Attenuates NF-κB binding to DNA	[77]
	LPS	Reduces LPS-induced nitric oxide, PGE_2_, TNF, IL-6, and IL-1β production	[82]
	*Staphylococcus aureus*	Reduces autophagy and phagocytosis while increasing NF-κB-mediated cytokine production	[90]
J774A.1 (Murine Macrophage Cell Line)	*Legionella pneumophila*	Prevents intracellular replication by reducing bacterial invasion	[86]
MH-S (Murine Macrophage Cell Line)	*Pseudomonas aeruginosa*	Blocks phagocytosis	[88]
Chick Microglial Cells	LPS	Inhibited nitric oxide production	[80]
BV2 (Microglial Cell Line)	LPS	Attenuates NF-κB binding to DNA	[76]
	LPS	Reduces LPS-induced NF-κB activity, nitric oxide, PGE_2_ IL-1β, and TNF production	[81]

## 5. Selected Experimentation

Since pneumonia—a common trigger of sepsis—served as the primary SARS-CoV-2 model in the recent landmark study on PI3k inhibition to mitigate severe infection-associated inflammatory damage [67], the companion findings in systemic MRSA infection inspired a subsequent series of experiments [91]. Our objective was to build upon these studies using a live bacterial model of MRSA and eganelisib (IPI549). We aimed to confirm the reduction in inflammatory markers that was observed in the murine model and to demonstrate a modest but statistically significant increase in immune cell function in human cells.

### 5.1. Bacterial Strains, Cell Lines, and Reagents Used

IPI549 was obtained through a materials transfer agreement (MTA) with the manufacturer Infinity Pharmaceuticals (Cambridge, MA, USA) and from Selleck Chemicals (Houston, TX, USA, #S8330). The doses used were verified to be non-bactericidal. The MRSA strain USA300-TCH1516 (ATCC, Manassas, VA, USA) was cultured in Todd–Hewitt broth (THB) at a 1:100 ratio to the mid-logarithmic phase and washed with PBS before use. The THP-1 human monocytic cell line (ATCC, Manassas, VA, USA, #TIB-202) was maintained in RPMI 1640 (ThermoFisher, Waltham, MA, USA, #11835-030) with 10% FBS (Cytiva, Marlborough, MA, USA, #SH30088.03), 4.5 g/L glucose (ThermoFisher, Waltham, MA, USA, #A2494001), 10 mM HEPES (ThermoFisher, Waltham, MA, USA, #15630-080), 1 mM sodium pyruvate (ThermoFisher, Waltham, MA, USA, #11360-070), and 0.05 mM 2-mercaptoethanol (ThermoFisher, Waltham, MA, USA, #21985-023). THP-1 cells were differentiated with 25 nM phorbol 12-myristate 13-acetate (PMA) (Sigma, St. Louis, MO, USA, #P1585) for 24 h before being washed with media for another 24 h before experiments. Immortalized murine macrophage wild-type and PIK3cg knockout cells, derived from mouse bone marrow and immortalized through J2 retrovirus transduction (in the labs of Judith Varner, UCSD, and Diana Hargreaves, Salk Institute) were cultured in DMEM with L-glutamine, glucose, and sodium pyruvate (Corning, Corning, NY, USA, #10013CV).

### 5.2. Statistical Analysis and Programs

Statistical analyses were performed with GraphPad Prism v10. Two-way ANOVA was used to compare murine wild-type and knockout macrophages, while one-way ANOVA and Student’s unpaired *t*-tests were used to compare significance across groups within one cell type. A *p*-value < 0.05 was considered statistically significant.

### 5.3. PI3k Knockout and Inhibition Reduces Inflammation in Human/Murine Macrophages

**Figure 2:**
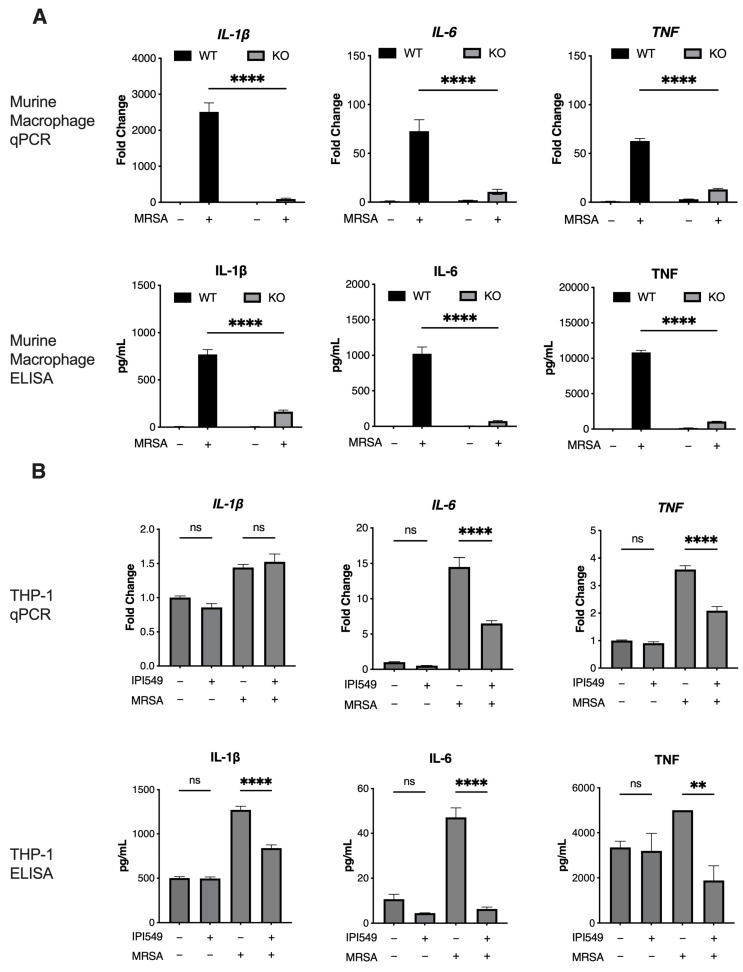
Pharmacological and genetic inhibition of PI3k suppresses inflammatory cytokine responses in both human and murine macrophages. Gene expression and ELISA quantified protein secretions of selected pro-inflammatory cytokines in (**A**) immortalized murine wild-type and knockout macrophages and (**B**) THP-1 cells treated with 1 µM IPI549 or vehicle, infected with MRSA (MOI of 1) for 4 h, and treated with 100 µg/mL gentamicin and 20 µg/mL lysostaphin 1 h post-infection. All data are representative of *n* = 3 independent experiments, with mean + SEM; ns = no significance, * *p* < 0.05, ** *p* < 0.01, *** *p* < 0.001, ****p < 0.0001.

Methods: Wild-type and knockout murine macrophages were plated at 500,000 cells per well in 24-well tissue culture plates (Corning, Corning, NY, USA, #3524) and infected with MRSA at a multiplicity of infection (MOI) of 1 for 4 h [92,93]. THP-1 cells were similarly plated and infected, with 1 µM IPI549 (or vehicle control) pretreatment for 1 h before infection, followed by 100 µg/mL gentamicin (MilliporeSigma, Burlington, MA, USA, #G1397) and 20 µg/mL lysostaphin (MilliporeSigma, Burlington, MA, USA, #L7386) 1 h post-infection to kill any remaining bacteria. RNA was isolated using Qiagen RNEasy Mini Kits (Qiagen, Hilden, Germany, #74104), reverse-transcribed, and analyzed by quantitative real-time PCR (qRT-PCR) with SYBR green on a BioRad CFX96 Touch Real-Time PCR Detection System (Bio-Rad Laboratories, Hercules, CA, USA). Gene expression was quantified using the ddCT method. Primer sequences are presented in Table S1. ELISAs were performed on harvested supernatants using mouse and human Duoset IL-1β/IL-1F2, IL-6, and TNF-α kits (R&D Systems, Minneapolis, MN, USA, #DY401, #DY206, #DY410, #DY201, #DY206, #DY210). Results: A decrease in IL-1β, IL-6, and TNF transcription and protein secretion was observed in both the knockout murine macrophages and IPI59-treated THP-1 cells upon MRSA infection (Figure 2A,B).

### 5.4. PI3k Knockout and Inhibition Reduces NF-κB Colocalization

**Figure 3:**
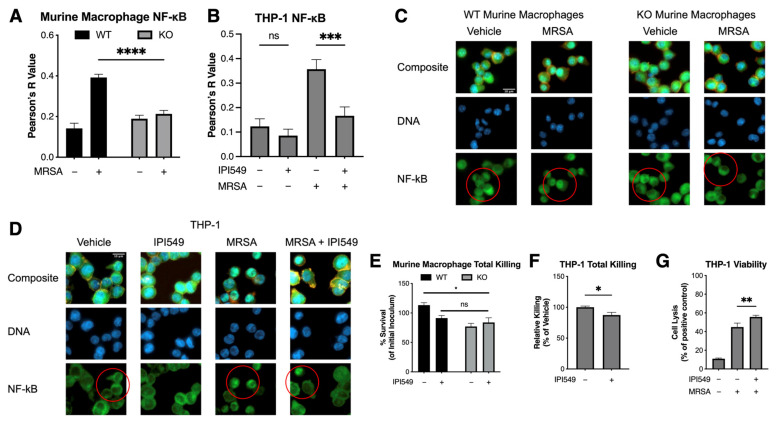
Pharmacological and genetic inhibition of PI3k reduces NF-κB translocation to the nucleus in both human and murine macrophages, without compromising bacterial killing ability. Pearson’s correlation values for NF-κB colocalization with DAPI as determined by pixel intensity correlation in (**A**) murine wild-type and knockout macrophages treated with IPI549 and MRSA supernatant and (**B**) THP-1 cells treated with IPI549 and MRSA supernatant. Representative images of NF-κB immunofluorescence from control and treated (**C**) wild-type and knockout murine macrophages and (**D**) THP-1 cells. NF-κB (in green) and DAPI (in blue). Images are 40× magnification. Bacterial survival in presence of (**E**) wild-type or knockout murine macrophages pretreated with vehicle or 1 µM IPI549 and (**F**) THP-1 cells pretreated with vehicle or 0.01 µM IPI549. (**G**) Lysis of THP-1 cells treated with vehicle, MRSA, or 0.01 µM IPI549, quantified by LDH release and normalized to 0.3% Saponin-positive control. All data are representative of *n* = 3 independent experiments, with mean + SEM; ns = no significance, * *p* < 0.05, ** *p* < 0.01, *** *p* < 0.001, **** *p* < 0.0001.

Methods: Wild-type, knockout murine macrophages and THP-1 cells were plated at 200,000 cells per well in a Nunc Lab-Tek II 8 Chamber Slide System (ThermoFisher, Waltham, MA, USA, #154534). THP-1 cells were pretreated with 1 µM IPI549 for 1 h. The MRSA supernatant, used due to bacterial autofluorescence, was isolated from an overnight culture, passed through a 0.22 µM filter, concentrated using a 3 kDa MWCO Amicon Ultra Centrifugal Filter (MilliporeSigma, Burlington, MA, USA, #UFC9003), diluted at 1:125, and added to the THP-1 cells for 1 h. The cells were fixed with 10% formalin, permeabilized with 0.25% Triton-X, and treated with an NF-κB p65 antibody (Cell Signaling Technology, Danver, MA, USA, #8242) and phalloidin-rhodamine (ThermoFisher, Waltham, MA, USA, #R415). After overnight incubation at 4 °C, the cells were exposed to a goat anti-rabbit IgG secondary antibody (ThermoFisher, Waltham, MA, USA, #A11070) and DAPI (ThermoFisher, Waltham, MA, USA, #D1306) and then mounted and imaged using a Zeiss Axio Observer.D1 Inverted Microscope (Zeiss, Dublin, OH, USA). Colocalization analysis was performed using ImageJ v2.14.0. For the murine macrophage images, the default automatic threshold was used to create an ROI using DAPI, and colocalization analyses of the DAPI and NF-κB channels were performed. For the THP-1 images, the “Huang” threshold was applied to the phalloidin-rhodamine channel alongside a rolling ball correction, before being merged with the DAPI channel. Image analysis macro commands are presented in Table S2. Results: Decreased intranuclear NF-κB was observed in both the knockout murine macrophages and IPI549-treated THP-1 cells, indicating reduced NF-κB translocation (Figure 3A–D).

### 5.5. Pharmacological and Genetic Inhibition of PI3kγ Does Not Compromise Bacterial Killing

Methods: Wild-type, knockout murine macrophages and THP-1 cells were plated at 100,000 cells per well in 96-well tissue culture plates, with the THP-1 cells being pretreated with 1 µM IPI549 or vehicle control for 1 h (Corning, Corning, NY, USA, #353072). Bacteria were grown as previously described, opsonized with pooled human serum at a 1:1 ratio for 5 min, and then incubated with cells at an MOI of 10 for 2 h. The cells were lysed with 0.3% saponin, incubated on ice for 5 min, diluted in a PBS, plated on Todd–Hewitt agar, and incubated overnight at 37 °C. Colony-forming units (CFUs) were enumerated the next day. The LDH content in supernatants from THP-1 cells treated with IPI549 and infected with MRSA was assessed using the LDH-Glo Cytotoxicity Assay kit (Promega, Madison, WI, USA, #J2380) and quantified with a Perkin Elmer EnSpire Alpha Multimode Plate Reader (Perkin Elmer, Shelton, CT, USA). Results: Slightly increased bacterial killing was observed in the knockout macrophages and IPI549-treated THP-1 cells, indicating that reduced inflammatory cytokines and NF-κB translocation did not compromise macrophage bacterial killing (Figure 3E,F). Increased cell lysis was observed with concurrent drug treatment and MRSA infection (Figure 3G).

## 6. Discussion and Areas of Future Study

**Figure 4:**
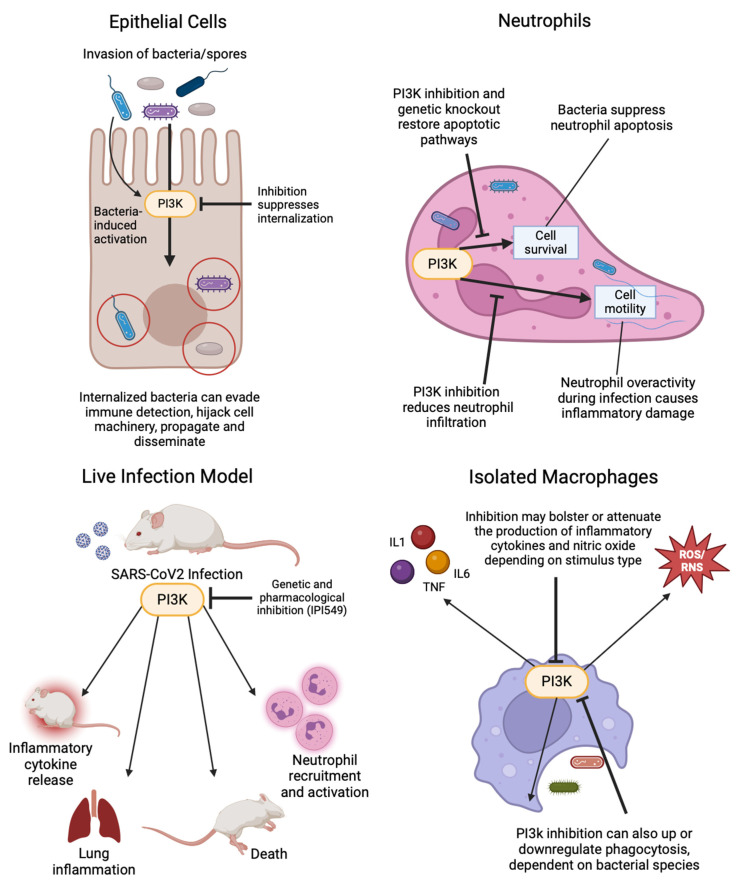
Schematic representation of the role of PI3k in infections of different cell types. The inhibition of PI3k can suppress the internalization of bacteria or spores in epithelial cells, restore apoptotic pathways, and reduce infiltration in neutrophils. In a live infection model, the inhibition of PI3k can reduce inflammatory cytokine release, lung inflammation, mortality, and neutrophil recruitment and activation. The inhibition of PI3k in macrophages may increase or decrease inflammatory cytokine release, nitric oxide production, and phagocytosis depending on stimulus type.

The PI3k pathway plays a central role in many types of cancers as a regulator of proliferation, metabolism, and survival, and it is also crucial for immune cell function [53,68,94,95,96,97]. This review focused on the broad effects of PI3k regulation across various cell types in response to bacterial infections (Figure 4). Although different pathogens elicit distinct responses, PI3k generally facilitates internalization and phagocytosis in both epithelial cells and phagocytes—processes that are suppressed by PI3k inhibition [47,50,84,86]. In neutrophils, many bacteria exploit the pro-survival effects of PI3k by upregulating anti-apoptotic proteins and NF-κB. This prevents the programmed cell death that is necessary to combat infection, allowing for intracellular replication to continue [55,56,57,58]. In macrophages, PI3k exhibits a paradoxical role: it can either enhance or reduce phagocytosis and modulate the inflammatory response, depending on the specific pathogen trigger [72,76,77,90,98]. The regulation of inflammation by natural products targeting the PI3k pathway further underscores the pathway’s significance [81,99,100,101]. Such findings in bacteria also raise questions about the role of PI3k in other pathogens, including viruses and parasites. As noted earlier, certain viral infections dysregulate PI3k, and its inhibition has been shown to restore normal apoptotic function or reduce viral replication [102,103]. Parasites, as eukaryotic or multicellular organisms, present more complex infection models, and significantly fewer studies have examined PI3k’s role in parasitic infections. However, the pharmacological inhibition of PI3k has shown promise in improving outcomes in *L. donovani* and *C. parvum* models [104,105]. Thus, PI3k still remains a compelling target not only for cancer therapeutics but for repurposing these drugs to treat infections.

Inflammation caused by infection is a major contributor to sepsis mortality, with a cascade of cytokines triggering an excessive immune response that leads to systemic organ damage and death [106]. Existing therapies aimed at controlling inflammation, such as steroids and cytokine-targeting antibodies, have shown limited success and often risk impairing immune cell function to the extent that infection prevails [107,108,109,110,111]. The recent discovery of PI3k’s role in modulating the inflammatory response during SARS-CoV-2 infection, particularly in vivo, prompted us to investigate whether this phenotype extends to human cells. In murine macrophages, knockout of the PIK3CG gene, which encodes the catalytic subunit of PI3kγ, significantly reduced inflammatory cytokine production. Similarly, in human THP-1 cells, treatment with the PI3kγ inhibitor IPI549 reduced inflammatory cytokine release. This suppression of cytokine expression and release in both murine macrophages and THP-1 cells was attributed to inhibited NF-κB translocation into the nucleus. Interestingly, these effects were accompanied by a modest but significant enhancement in bacterial killing activity and an increase in cell lysis. Although the cell viability decreased, the improvement in bacterial clearance is noteworthy, as programmed cell death can enhance pathogen elimination [112,113,114]. While the concentrations of the PI3k inhibitor were not directly bactericidal, the ability to modulate the immune response offers a valuable therapeutic approach. These results align with PI3k’s generally pro-inflammatory role in bacterial infection. However, these studies are among the first to utilize bacterial supernatant as a stimulus, suggesting that the role of PI3k in inflammation may depend on specific physiological signals—whether originating from the host, the bacteria itself, or secreted virulence factors and other stimuli.

Our studies, along with those of Sheppard and Ghebremedhin et al. [67], confirm that the PI3kγ isoform plays a significant role in infection-induced inflammation, complementing its well-established function in cancer. This inflammatory role can be effectively modulated through genetic or pharmacological inhibition, potentially providing an advantage in combating bacterial infections. Given PI3k’s extensive role in regulating both proliferative and immune functions across various cell types, it remains a compelling target for further investigation. Future research should focus on developing novel chemical scaffolds to modulate the enzyme and on better defining its role in the context of infection.

## Figures and Tables

**Table 1:** PI3k inhibitors that are FDA-approved or under current investigation in clinical trials.

Drug Name	Class	Mode of Action	Clinical Trial Stage	Reference
Alpelisib	PI3k-α inhibitor	Selectively targets the mutated PI3k-α in many solid tumors to suppress increased activity	Approved	[25,26]
Copanlisib	Pan-PI3k inhibitor	Targets all class I PI3k isoforms to hinder B-cell proliferation and survival in follicular lymphomas	Approved	[25,27]
Duvelisib	PI3k-γ, δ inhibitor	Selectivity for γ and δ isoforms in treatment of CLL and inflammatory and autoimmune conditions	Approved	[25,28]
Idelalisib	PI3k-δ inhibitor	Inhibits δ isoform in hematopoietic cells to slow B-cell cancer proliferation	Approved	[25,29]
Umbralisib	PI3k-δ inhibitor	Inhibits δ isoform and casein kinase 1ε in treatment of CLL and other lymphomas	Approved	[25,30]
TL117	Pan-PI3k inhibitor	Combination therapy with paclitaxel to treat head and neck squamous cell carcinoma	I/II	NCT04843098, [25]
GSK2636771	PI3k-β	Blocks β isoform to treat cancers with PTEN mutations	II	NCT04439149
Eganelisib (IPI-549)	PI3k-γ inhibitor	Used in combination with Tecentriq and Abraxane to treat triple-negative breast cancer or with Tecentriq and Avastin to treat renal cell carcinoma	II	NCT03961698
AZD8186	PI3k-β inhibitor	Combination therapy with docetaxel to treat solid tumors with PTEN or PIK3-β mutations	I	NCT03218826

## Data Availability

All data generated or analyzed during this study are included in this published article. Additional data are available from the corresponding author on reasonable request.

## Supplementary Materials

The following supporting information can be downloaded at https://www.mdpi.com/article/10.3390/antibiotics14030315/s1: Table S1: Primer sequences; Table S2. ImageJ Analysis Macro Commands.
