# Supplementary material for: The Role of PI3k-Gamma Modulation in Bacterial Infection: A Review of the Literature and Selected Experimental Observations
Source: Antibiotics (Basel). 2025 Mar 18;14(3):315. doi: 10.3390/antibiotics14030315 (PMC11939471; doi:10.3390/antibiotics14030315)
Supplement: Supplementary file 1 [file antibiotics-14-00315-s001.zip › antibiotics-3413156-supplementary.pdf]

Table S1. Primer sequences:.

Mouse IL1B FOR:	AACTCAACTGTGAAATGCCACC
Mouse IL1B REV:	CATCAGGACAGCCCAGGTC
Mouse IL6 FOR:	TACTCGGCAAACCTAGTGCG
Mouse IL6 REV:	GTGTCCCAACATTCATATTGTCAG T
Mouse TNFa FOR:	CACCACGCTCTTCTGTCTACTG
Mouse TNFa REV:	GCTACAGGCTTGTCACTCGAA
Human IL1 FOR:	TCTGTACCTGTCCTGCGTGT
Human IL1 REV:	ACTGGGCAGACTCAAATTCC
Human IL6 FOR:	GATGAGTACAAAAGTCCTGATCC A
Human IL6 REV:	CTGCAGCCACTGGTTCTGT
Human TNFa FOR:	CAGCCTCTTCTCCTTCCTGAT
Human TNFa REV:	GCCAGAGGGCTGATTAGAGA

Table S2. ImageJ Analysis Macro Commands:.

Figure 3A:

```
Title= getTitle();
run("Stack to Images");
close();
rename(Title + "dapi");
run("Put Behind [tab]");
rename(Title + "nfkb");
selectWindow(Title + "nfkb");
run("Duplicate...", " ");
rename("Duplicate");
setAutoThreshold("Default dark");
run("Create Selection");
roiManager("Add");
close("Duplicate");
run("Coloc 2");
roiManager("select", 0);
roiManager("delete");
selectWindow(Title + "nfkb");
close();
selectWindow(Title + "dapi");
```

Figure 3B:

```
Title= getTitle();
```

 https://doi.org/10.3390/xxxxx

```
run("Stack to Images");
rename("rhoda");
run("Put Behind [tab]");
rename("nfkb");
run("Put Behind [tab]");
rename("dapi");
selectWindow("dapi");
run("Duplicate...", " ");
rename("dapiDuplicate");
selectWindow("rhoda");
setAutoThreshold("Huang dark no-reset");
setOption("BlackBackground", true);
run("Convert to Mask");
selectWindow("dapiDuplicate");
run("Subtract Background...", "rolling=50");
setAutoThreshold("Default dark no-reset");
run("Convert to Mask");
imageCalculator("Add create", "dapiDuplicate","rhoda");
rename("merge");
selectWindow("merge");
run("Erode");
run("Erode");
run("Create Selection");
roiManager("Add");
roiManager("Select", 0);
roiManager("Rename", "ROI");
selectWindow("nfkb");
rename(Title + "nfkb");
selectWindow("dapi");
rename(Title + "dapi");
run("Coloc 2");
roiManager("select", 0);
roiManager("delete");
selectWindow(Title + "nfkb");
close();
selectWindow(Title + "dapi");
close();
selectWindow("rhoda");
close();
```

```
selectWindow("dapiDuplicate");
close();
selectWindow("merge");
close();
```
